# Display of the Viral Epitopes on *Lactococcus lactis*: A Model for Food Grade Vaccine against EV71

**DOI:** 10.1155/2013/431315

**Published:** 2013-02-13

**Authors:** Nadimpalli Ravi S. Varma, Haryanti Toosa, Hooi Ling Foo, Noorjahan Banu Mohamed Alitheen, Mariana Nor Shamsudin, Ali S. Arbab, Khatijah Yusoff, Raha Abdul Rahim

**Affiliations:** ^1^Cellular and Molecular Imaging Laboratory, Department of Radiology, Henry Ford Hospital, Detroit, MI 48202, USA; ^2^Institute of Bioscience, Faculty of Biotechnology and Biomolecular Sciences, Universiti Putra Malaysia, 43400 Serdang, Selangor, Malaysia; ^3^Department of Bioprocess Technology, Faculty of Biotechnology and Biomolecular Sciences, Universiti Putra Malaysia, 43400 Serdang, Selangor, Malaysia; ^4^Department of Cell and Molecular Biology, Faculty of Biotechnology and Biomolecular Sciences, Universiti Putra Malaysia, 43400 Serdang, Selangor, Malaysia; ^5^Department of Medical Microbiology and Parasitology, Faculty of Medicine and Health Sciences, 43400 Serdang, Selangor, Malaysia; ^6^Department of Microbiology, Faculty of Biotechnology and Biomolecular Sciences, Universiti Putra Malaysia, 43400 Serdang, Selangor, Malaysia

## Abstract

In this study, we have developed a system for display of antigens of Enterovirus type 71 (EV71) on the cell surface of *L. lactis*. The viral capsid protein (VP1) gene from a local viral isolate was utilized as the candidate vaccine for the development of oral live vaccines against EV71 using *L. lactis* as a carrier. We expressed fusion proteins in *E. coli* and purified fusion proteins were incubated with *L. lactis*. We confirmed that mice orally fed with *L. lactis* displaying these fusion proteins on its surface were able to mount an immune response against the epitopes of EV71. This is the first example of an EV71 antigen displayed on the surface of a food grade organism and opens a new perspective for alternative vaccine strategies against the EV71. We believe that the method of protein docking utilized in this study will allow for more flexible presentations of short peptides and proteins on the surface of *L. lactis* to be useful as a delivery vehicle.

## 1. Introduction 

Enterovirus 71 infection manifests most frequently as the childhood illness known as hand-foot- and-mouth disease (HFMD) and is considered to be clinically indistinguishable from HFMD caused by Coxsackie A16 (CA16). However, the former has the propensity to cause neurological disease during acute infection, a feature not observed in CA16 infections [1]. Children under 5 years of age are partichltularly susceptible to the more severe forms of EV71-associated neurological disease, including aseptic meningitis, brainstem or cerebellar encephalitis, and acute flaccid paralysis. Several large epidemics of severe EV71 infection in young children, including numerous cases of fatal brainstem encephalitis, have recently been reported in South East Asia and Western Australia [2–6] raising concern that there may be an increase in both the prevalence and virulence of EV71. Two candidate vaccines against EV71 utilizing a formalin-inactivated whole virus and a DNA vaccine expressing VP1 have previously been developed [7]. In addition, both recombinant and subunit vaccine strategies optimized as a neutralizing antibody had been shown to provide some protection against EV71 lethal challenges in neonatal mice [8].

The use of a live, food grade organism that is noninvasive and nonpathogenic as antigen delivery vehicle is a promising vaccine strategy. This strategy could overcome potential problems due to the use of live attenuated enteroviral strains, which may have the risk of reversion and residual virulence. The immunogenicity by *L. lactis* expressing several bacterial and viral antigens has been documented [9–11]. One of the main factors inhibiting their use in a live vaccine delivery is the lack of expression vectors with strong promoters. To overcome these problems associated with high expression of proteins in *L. lactis*, we have chosen the *E. coli* expression host due to the availability of a wide variety of expression vectors and that recombinant proteins produced in *E. coli* can be easily purified. In this work, we expressed and purified individually the fusion proteins (viral epitopes fused with cell wall binding anchor protein) and successfully anchored the epitopes on the outer surface of *L. lactis *to be presented as a surface displayed antigen. Preliminary immunological studies have demonstrated the generation of specific antibody responses in mice orally fed with *L. lactis* displaying epitopes of EV71.

## 2. Materials and Methods

### 2.1. Microorganisms

*Escherichia coli* TOP10 (Invitrogen, Carlsbad, CA, USA) was used as a cloning host. *E. coli *BL21 (DE3) F^−^ *omp*T *hsd*S_B_  (r_B_^−^m_B_^−^)  *gal dcm *(DE3) plysS (Cam^R^) was used as the *E. coli *expression host. *L. lactis *MG1363 [12], was used to display the viral epitopes.

### 2.2. Culture Conditions

Lactococcal cells were grown at 30°C in M17 broth (Oxoid, USA) (Tryptone (5 g/L), Soya peptone (5 g/L), Lab-Lemco (5 g/L), Yeast extract (2.5 g/L), Ascorbic acid (0.5 g/L), Magnesium sulphate (0.5 g/L), and Di-sodium-glycerophosphate (19 g/L)) or M17 agar with 0.5% glucose as standing culture. *E. coli *cells were grown at 37°C with agitation in Luria-Bertani (LB) (Oxoid) broth (Tryptone (10 g/L), Yeast extract (5 g/L), and NaCl (10 g/L)). Whenever required, a total concentration of 50 *μ*g/mL ampicillin was used for the recombinant *E. coli *cultures.

### 2.3. Plasmids

pCR 2.1 (Amp^R^, Km^R^, Invitrogen) is an *E. coli *vector used for subcloning the nucleotides from 1 to 201 (VP1_1-201nt_) and 103 to 300 (VP1_103-300nt_) of VP1 gene of EV71; pSVac (Amp^R^) is an *E. coli* expression plasmid harbouring the N-acetylmuramidase (*acmA'*) gene fragment [13]. pRSETC (Amp^R^, Invitrogen) is pUC-derived expression vector designed for high level protein expression of cloned genes under T7 promoter in *E. coli*.

### 2.4. Construction of Plasmids pSVacVP1_1-201_ and pSVacVP1_103-300_

Plasmids pSVacVP1_1-201_ and pSVacVP1_103-300_ were previously constructed [13]. The N-terminal region A1 represents VP1_1-201_ and N-terminal region A3 represents VP1_103-300_. 

### 2.5. Expression Studies

The *E. coli *BL21 (DE3) plysS cells containing the recombinant vectors were grown overnight at 37°C with shaking at 250 rpm. The cells were subcultured into a fresh 10 mL of LB medium containing ampicillin (50 *μ*g/mL) and chloramphenicol (35 *μ*g/mL) grown to an OD_600_ of 0.6 before being induced with 1 mM of isopropyl-*β*-D-thiogalactopyranoside (IPTG) for 3 h. Sodium dodecyl sulfate-polyacrylamide gel electrophoresis (SDS-PAGE) was performed according to Laemmli, 1970 [14], using 10%–12.5% (w/v) polyacrylamide gels. The *E. coli *cultures were harvested by centrifugation at 2,000 g for 10 min. The cell pellets were resuspended in 100 *μ*L of 2X sample buffer (0.125 M Tris, 4% SDS, 0.2 M DDT, 0.02% bromophenol blue, and 20% glycerol) prior to boiling at 95°C for 5 min before centrifugation at 10,000 g. A volume of 10 *μ*L of the supernatant was loaded onto the gel. Semidry blotter (Hoeffer, Pharmacia Biotech, UK) was utilized to transfer the electrophoresed protein bands from the SDS-PAGE to polyvinylidene difluoride (PVDF) membrane. The membrane was then incubated in 1% (w/v) blocking solution (Roche Diagnostics GmbH, Mannheim, Germany) for 1 h at room temperature with gentle agitation. Then the membrane was incubated with primary rabbit anti-VP1 (Professor Dr. Mary Jane Cardosa, Universiti Malaysia Sarawak, Malaysia). The conjugated membrane was then washed with TSBT (Roche) three times before incubation with peroxidase-labelled goat anti-rabbit IgG secondary antibody (50 mU/mL in 0.5% (w/v) blocking solution (Roche)) for 60 min at room temperature with gentle agitation. After washing with TSBT, the membrane was exposed to film for 20 min and visualized.

### 2.6. Purification of Recombinant Protein Fragment and Binding to *L. lactis*

The cell cultures (10 mL) were harvested after 2-3 h induction with IPTG. The cells were resuspended in 400 *μ*L of PBS pH 7.4 (20 mM K_2_HPO_4_, 5 mM KH_2_PO_4_, and 150 mM NaCl) and then lysed by a combination of lysozyme (10 mg/mL) and glass beads (Sigma, St. Louis, MO, USA). The crude homogenates were centrifuged at 10,000 g for 15 min and the supernatant was applied into the Ni^2+^ affinity column (Qiagen GmbH, Germany). The recombinant proteins were eluted with 250 mM imidazole buffer and each of their concentrations calculated based on Bradford method using the Bio-Rad protein assay kit (Bio-Rad, USA). Three mL of exponentially grown *L. lactis *MG1363 were centrifuged and gently resuspended in 600 *μ*L of fresh M17 broth. Then, 200 *μ*L of purified AcmA/VP1_1-67aa_ and AcmA/VP1_35-100aa_ were separately added to 600 *μ*L of the cells and incubated at 30°C for 2 h. The mixture was then centrifuged again at 2,000 g for 10 min and the cell pellets were washed with 1 mL of PBS three times. The binding of the purified recombinant proteins were then analysed by immunofluorescence microscopy.

### 2.7. Immunofluorescence Microscopy

The control *L. lactis *cells and cells mixed with either the AcmA/VP1_1-67aa_ or AcmA/VP1_35-100aa_ fusion proteins were initially placed on chamber slides precoated with poly-L-lysine followed by incubation for 15 min before being fixed with 4% paraformaldehyde. Cells were also incubated with 3% bovine serum albumin (BSA) in PBS for 30 min at room temperature to block nonspecific binding and washed with PBS. The fixed cells were then labeled with primary (rabbit anti-VP1) antibodies. The slide was then washed with PBS and incubated with rhodamine labeled goat anti-rabbit secondary antibody (diluted at 1 : 200 in 1% BSA) at room temperature for 1 h. This was followed by washing with PBS three times, air-dried, and mounted in an antifading agent (Fluoroguard, (Bio-Rad, Hercules, CA, USA)). The labeled slides were then analysed by Confocal Microscope (Bio-Rad MRC 1024 Confocal Laser Scanning Microscope, Bio-Rad) (excitation 550 nm; emission 570 nm). Cells were observed under a 40x objective. Images were taken and analyzed with Bio-Rad laser sharp software (Bio-Rad).

### 2.8. Stability Assay

Stability of anchored protein on cell surface was analyzed for a period of 5 days. In brief, *L. lactis* cells were added to fusion protein and incubated at 30°C for 2 h. The mixture was centrifuged and washed with PBS. ELISA was carried out on the *L. lactis* cells displaying fusion protein at every 24 h up to 120 h to determine the stability. The lithium chloride stability assay was performed to further test the stability of the anchored proteins [15].* L. lactis* cells incubated with fusion proteins were harvested and treated with 100 *μ*L of 8 M LiCl solution at 30°C for 30 min. After treatment, cells were analyzed by ELISA for the detection of the presence of fusion proteins on the cell surface of *L. lactis*.

### 2.9. Immunogenicity Studies

Specific pathogen-free female 2-week-old BALB/c mice were used. The mice were housed in microisolator cages with free access to water and feed. Three groups of 5 mice were orally fed with 500 *μ*L of *L. lactis *cells (10^9^ cells) displaying either AcmA/VP1_1-67aa_ fusion protein or AcmA/VP1_35-100aa_ fusion protein or with both of the fusion proteins. The first control mice group was immunized with the 500 *μ*L (10^9^ cells) of *L. lactis *cells. The second control mice group received 500 *μ*L of PBS. All the mice were fed using oral gavage tube without anesthesia and received the booster dose (same as the initial immunization dose) on days 7, 14, and 21. Blood samples were collected from a tail vein of the immunized mice at 0, 7, 14, 21, 28, and 35 days, and the collected blood was incubated at 37°C for 1 h. The sera were separated from red blood cells by centrifugation at 4,500 g for 10 min and stored at 4°C. For long term storage, serum samples were kept at −20°C. 

### 2.10. Western Blot for the Detection of Antigen-Specific Serum Antibody

Purified fusion proteins (AcmA/VP1_1-67aa_ and AcmA/VP1_35-100aa_) and total protein extractions of *L. lactis *and *E. coli* BL21 (DE3) pLysS (pRSETC) cells were separated by 12.5% SDS-PAGE and electroblotted on a PVDF (Millipore Corp., Billerica, MA, USA) membrane. The membrane was then incubated in 1% (w/v) BSA in DBT (Amresco, Solon, OH, USA) for 1 h, followed by incubation for 1 h in 10 mL of DBT (Amresco) containing 10 *μ*L of the respective sera collected at day 21 (7 days after the 2nd booster dose) from the immunized mice. After washing with the DBT, the conjugated membrane was incubated with goat anti-mouse antibody conjugated HRP (50 mU/mL in TBS, (Amresco) for 1 h, washed with DBT, and developed using 4-chloronaphthol (Amresco).

### 2.11. Analysis of Antigen-Specific Serum Antibody by ELISA

ELISA plate wells were coated with purified recombinant VP1 protein (complete VP1 protein; 1 *μ*g/mL in coating buffer 0.015 M Na_2_CO_3_, 0.03 M NaHCO_3_, and pH 9.6). ELISA plates coated with EV71 virus were also used to analyse the serum of immunized mice. (The purified recombinant VP1 protein and EV71 virus coated plates were obtained from Professor Dr. Mary Jane Cardosa, Universiti Malaysia Sarawak, Malaysia). The wells were blocked with 2% BSA in PBS for 1 h. A volume of 100 *μ*L of serially diluted hyperimmune mouse sera (1 : 1000; 1 : 10,000, and 1 : 100,000 dilutions) were added to the wells and incubated for 1 h. The serum from blood collected at 0, 7, 14, 21, and 28 days from all groups of immunized mice was analyzed at 1 : 1000; 1 : 10,000, and 1 : 100,000 dilutions. The wells were then washed six times with 1x PBS before incubation with the secondary antibody (100 *μ*L of HRP conjugated anti-rabbit antibodies (Roche, Switzerland) diluted at 1 : 500 in 0.5% BSA in 1x PBS) at room temperature for 1 h. After incubation, the unconjugated secondary antibody was removed by washing with 1x PBS (6 times, 10 min each). Then, 100 *μ*L of substrate (BM Blue, Roche) was added to each well. After color development the reaction was stopped by adding 50 *μ*L of 1 M H_2_SO_4_ and absorbance was measured by ELISA reader at OD_450_.

## 3. Results

### 3.1. Construction of pSVacVP1_1-201nt_, pSVacVP1_103-300nt_ and Expression of AcmA/VP1_1-67aa_ and AcmA/VP1_35-100aa_

**Figure 1:**
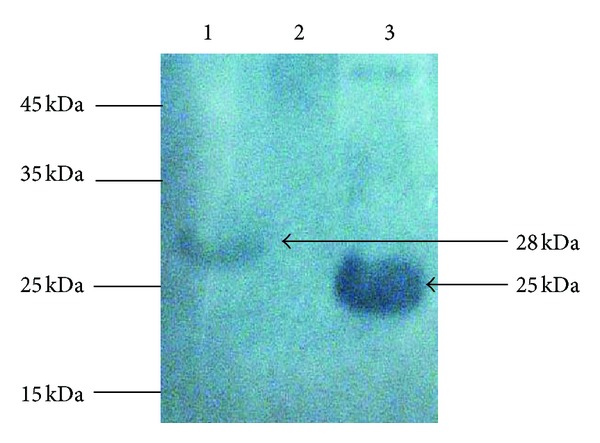
Western blot analyses of the over-expressed recombinant fusion proteins (AcmA/VP1_1-67aa_ and AcmA/VP1_35-100aa_). Lane 1, Total protein of BL21 (DE3) pLysS (pSVacVP1_1-201nt_); lane 2, Total protein of BL21 (DE3) pLysS (pRSETC) as negative control; lane 3, Total protein of BL21 (DE3) pLysS (pSVacVP1_103-300nt_). The arrow shows recombinant fusion proteins: AcmA/VP1_1-67aa_ (~28 kDa), AcmA/VP1_35-100aa_ (~25 kDa).

Two fragments of the N-terminal region of VP1 were amplified and subcloned separately into plasmid pSVac [13]. Total protein extracts of *E. coli *BL2 (DE3) containing the recombinants AcmA/VP1_1-67aa_ and AcmA/VP1_35-100aa_ and *E. coli *BL21 (DE3) were analyzed by SDS-PAGE and Western blot. The SDS-PAGE protein profile showed the presence of 28 kDa and 25 kDa bands, which approximately corresponded to the expected size of the recombinant AcmA/VP1_1-67aa_ and AcmA/VP1_35-100aa_. Western blot analysis using anti-VP1 confirmed that the two bands were immunoreactive to the antibody (Figure 1). This suggested that the pSVacVP1_1-201nt_ and pSVacVP1_103-300nt_ recombinant constructs were successfully expressed in *E. coli*.

### 3.2. Affinity Purification of Recombinant Fusion Proteins

In order to study the display of EV71 capsid protein (VP1_1-201nt_ and VP1_103-300nt_ regions of VP1 gene) on the cell wall surface of *L. lactis*, recombinant *E. coli* BL21 (DE3) pLysS cells harbouring pSVacmVP1_1-201_, pSVacmVP1_103-300_, pSVnpVP1_1-201_, and pSVnpVP1_103-300_ vectors were grown and induced with IPTG (Gibco BRL, USA). The protein fractions from the cells were purified on Ni^2+^affinity columns, and the eluted proteins were analysed by SDS-PAGE (data not shown).

### 3.3. Binding of the EV71 VP1 Epitopes to the Cell Surface of *Lactococcus*

**Figure 2:**
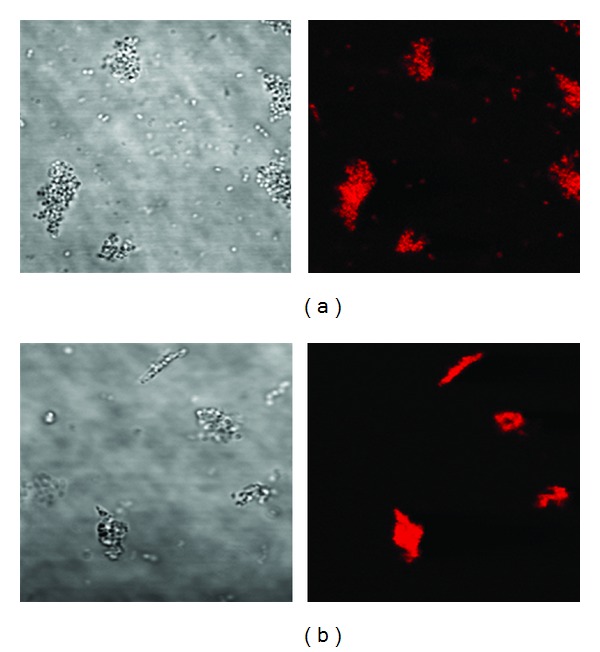
Confocal micrographs of the binding of fusion proteins to *L. lactis*: (a) bright field and fluorescence image of *L. lactis *cells incubated with AcmA/VP1_1-67aa_ protein; (b) bright field and fluorescence image of *L. lactis *cells incubated with AcmA/VP1_35-100aa_ protein.

Purified AcmA/VP1_1-67aa_ and AcmA/VP1_35-100aa_ fragments were incubated with *L. lactis *and subjected to ELISA analysis and immunofluorescence staining. A positive color change was detected for the *L. lactis *cells incubated with AcmA/VP1_1-67aa_, AcmA/VP1_35-100aa_ fusion proteins. Immunofluoresence analysis also indicated that the display of AcmA/VP1_1-67aa_ and AcmA/VP1_35-100aa_ fusion proteins on the *L. lactis *cell surface was in stable conformation. It was observed that the *L. lactis *cells incubated with both fragments (AcmA/VP1_1-67aa_ and AcmA/VP1_35-100aa_) were efficiently stained by rhodamine labeled secondary antibody whilst the control cells remained free from staining (Figure 2). These results strongly suggest that the fusion proteins constituting the AcmA/VP1_1-67aa_ and AcmA/VP1_35-100aa_ expressed in *E. coli *had maintained the active binding domains and the capacity to dock-onto the outer surface of *L. lactis *cell wall. 

### 3.4. Binding Stability of Fusion Proteins on the Surface of *L. lactis*

In order to apply this system for the display of foreign proteins on *L. lactis*, it is important to determine the stability of the anchorage of fusion proteins. The stability assay was conducted for 5 days, at each 24 h interval, after which the *L. lactis *cells incubated with the fusion proteins (AcmA/VP1_1-67aa_ and AcmA/VP1_35-100aa_) were probed with rabbit anti-VP1 antibody. This was followed by HRP conjugated anti-rabbit IgG antibody (Roche) before being analysed by ELISA reader.* L. lactis *cells without incubation with fusion proteins were used as the negative control. The fusion proteins still present on the surface of *L. lactis* even after five days of incubation (data not shown). We further tested stability of anchored protein by treating with LiCl. LiCl is commonly used to remove proteins from bacterial cell walls. We interested to observe the effect of LiCl on *L. lactis* cells displaying AcmA/VP1_1-67aa_ or VP1_35-100aa_. The mode of action of LiCl is the cleavage of covalent or noncovalent bonds between the surface proteins and cell walls. We want to test the stability of anchored proteins by treating LiCl. *L. lactis* displaying fusion proteins (AcmA/VP1_1-67aa_ and AcmA/VP1_35-100aa_) were treated with 8 M LiCl, after the treatment of cells was analyzed by whole cell ELISA. Results showed the presence of fusion proteins on the cell surface of *L. lactis* even after treatment with LiCl, which indicates that the proteins are anchored strongly to the cell surface (data not shown). 

### 3.5. Detection of Serum Antibody Response for VP1_1-67aa_ and VP1_35-100aa_ of VP1 in Mice

**Figure 3:**
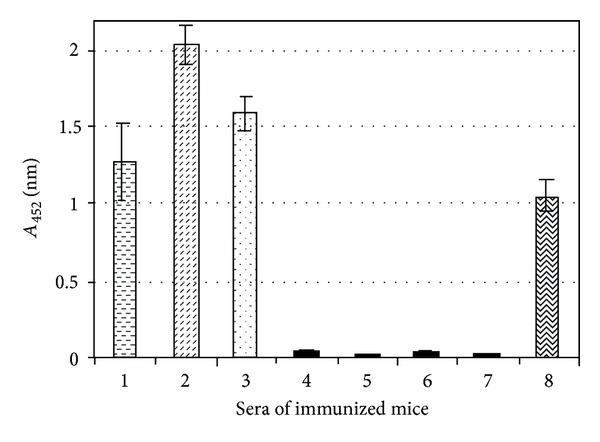
Analysis of serum from immunized mice using VP1 coated ELISA plates. 1: serum of mice immunized with *L. lactis* displaying VP1_1-67aa_; 2: serum of mice immunized with *L. lactis* displaying VP1_1-67aa_ and VP1_35-100aa_; 3: serum of mice immunized with *L. lactis* displaying VP1_35-100aa_; 4: serum of control mice immunized with *L. lactis*; 5: serum of control mice immunized with PBS; 6: serum of the rabbit; 7: serum of preimmunized mice; 8: rabbit anti-VP1 antibodies as positive control. Sera from immunized mice (Balb/c) were taken after 3rd booster immunization_._ Testing of sera was done at sera dilution 1 : 10,000. Note: the absorbance value shown was after the deduction of the background value obtained from purified His-tag protein.

The sera of mice orally immunized with live *L. lactis *cells displaying VP1_1-67aa_ or VP1_35-100aa_ antigens were tested for VP1 specific antibodies by ELISA using purified recombinant VP1 fusion protein (complete VP1 protein) as the antigen. The antiserum from mice orally fed with *L. lactis* displaying the immunogens (VP1_1-67aa_ or VP1_35-100aa_ or both) clearly reacted with the fusion proteins (recombinant VP1  fusion protein of EV71) (Figure 3), whereas the antiserum from mice orally immunized with only *L. lactis* or mice orally immunized with PBS did not react with the recombinant VP1 fusion protein of EV71 (Figure 3). 

**Figure 4:**
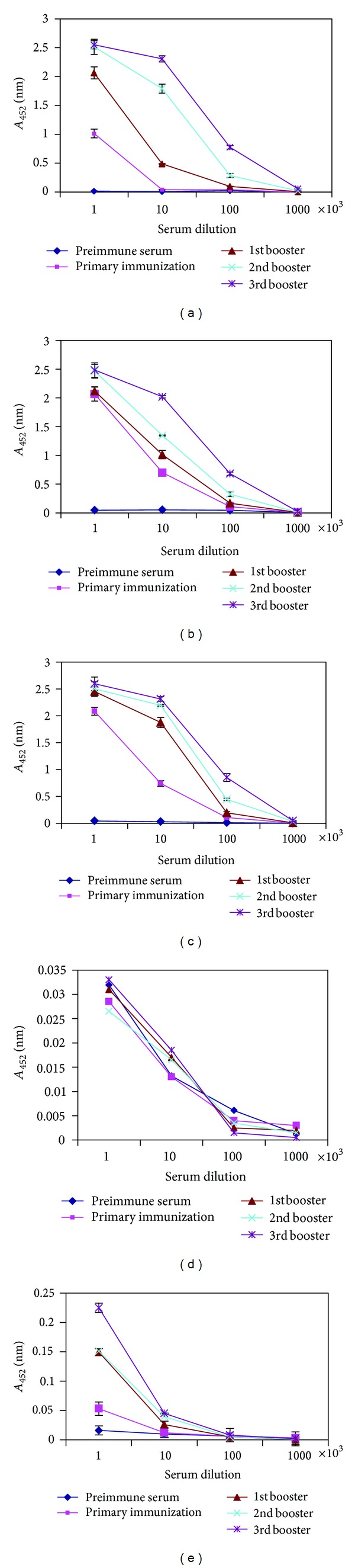
Determination of antibody titers by ELISA. (a) Serum from mice immunized with *L. lactis* displaying VP1_1-67aa_, (b) serum from mice immunized with *L. lactis* displaying VP1_35-100aa_, (c) serum from mice immunized with *L. lactis* displaying both epitopes (VP1_1-67aa_ and VP1_35-100aa_), and (d) serum from control mice immunized with PBS. (e) Serum from control mice immunized with *L. lactis*. Antibodies were measured using complete VP1 protein coated ELISA plates. Sera from mice (Balb/c) were taken before and after each immunization with *L. lactis* displaying VP1_1-67aa_.

The antibody titers of mice orally fed with *L. lactis* displaying VP1_1-67aa_ were shown to have lower antibody titers after primary immunization when compared with the antibody titers of mice orally fed with *L. lactis* displaying AcmA/VP1_1-67aa_ or VP1_35-100aa_ (Figure 4(a)). The antibody titers increased after the 1st booster dose in *L. lactis* displaying VP1_1-67aa_ (Figure 4(a)). On the other hand, mice orally fed with *L. lactis* displaying VP1_35-100aa_ gave a higher level of antibody titers in primary immunized serum as well as in all booster doses when compared to the antibody titers of mice fed with *L. lactis* displaying VP1_1-67aa_ (Figure 4(b)). The highest level of antibody titers at 1 : 1000 dilution was, however, seen in the serum of mice fed with *L. lactis* displaying both epitopes when compared to* L. lactis* displaying only VP1_1-67aa_ or VP1_35-100aa_ (Figure 4(c))_._ These results indicated a better response when a combination of both epitopes were used. There was no reaction between recombinant VP1 fusion protein and the serum of mice orally immunized with PBS (Figure 4(d)). A very minor reaction was observed with the serum of mice immunized with *L. lactis* at 1 : 1000 serum dilution and lower (Figure 4(e)). 

**Figure 5:**
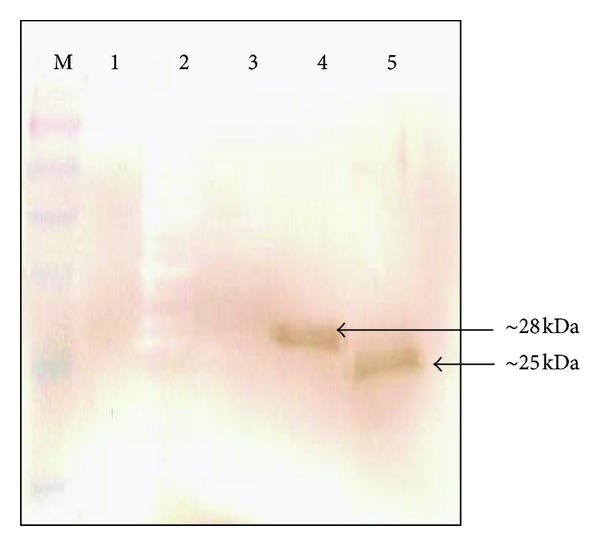
Detection of serum antibody response against VP1_1-67aa_ and VP1_35-100aa_ epitopes of EV71 in mice immunized with *L. lactis *displaying AcmA/VP1_35-100aa_. Lane 1: BSA protein; lane 2: *E. coli *(pRSET) total proteins; lane 3: total proteins of *L. lactis* MG1363; lane 4: purified AcmA/VP1_1-67aa_ proteins; lane 5: purified AcmA/VP1_35-100aa_ protein; lane M: protein marker (Fermentas, Hanover, MD, USA). The arrow shows recombinant fusion proteins: AcmA/VP1_1-67aa_ (~28 kDa) and AcmA/VP1_35-100aa_ (~25 kDa).

In addition, ELISA results demonstrated that the antiserum from mice orally fed with *L. lactis* displaying immunogens (VP1_1-67aa_ or VP1_35-100aa_ or both) of VP1 of EV71 clearly reacted in the wells coated with EV71 virus (data not shown), whereas the antiserum from mice orally fed with only *L. lactis* or mice orally given PBS did not react with the EV71 virus (data not shown). These results clearly indicated that the fusion proteins (AcmA/VP1_1-67aa_ and AcmA/VP1_35-100aa_) displayed on the cell surface of *L. lactis* were able to elicit an antigen-specific immune response in mice against VP1 protein. The antibody response against VP1_1-67aa_ and VP1_35-100aa_ antigens of EV71 in mice was also tested by Western blot analysis. Groups of five mice were orally immunized with live *L. lactis *cells displaying AcmA/VP1_1-67aa_ and AcmA/VP1_35-100aa_ and mouse sera (7 days after the second booster dose) were tested for AcmA/VP1_1-67aa_ and AcmA/VP1_35-100aa_ specific antibodies by Western blot analysis using AcmA/VP1_1-67aa_ and AcmA/VP1_35-100aa_ fusion proteins as the capturing antigens. The antisera from mice orally fed with *L. lactis *displaying either one or both of the fusion proteins of EV71 were shown to have reacted with the fusion proteins (Figure 5), whereas the antisera from mice orally fed with only *L. lactis *or PBS did not show any positive reaction (data not shown). These results clearly indicate that the fusion proteins (AcmA/VP1_1-67aa_ and AcmA/VP1_35-100aa_) displayed on the cell surface of *L. lactis *were able to elicit an antigen-specific immune response in the mice.

## 4. Discussion 

A system for targeting purified anchor proteins to the cell surface of *Lactococcus *and other lactic acid bacteria (LAB) has been developed [13, 15, 16]. Since *L. lactis *is a noncolonizing commensal organism, the approach of this work was to append the surface of the organism *in vitro *with antigens prior to immunization to enhance antibody response. Our objectives were to study the capability of the purified anchor protein AcmA that has gone through the *E. coli* system to attach and deliver specific antigens such as those of VP1_1-67aa_ and VP1_35-100aa_ fragments onto the surface of *L. lactis *in order to elicit an immune response in the host. *L. lactis *has been reported to successfully express and target tetanus toxin model antigen into the cytoplasm, cell wall, and extracellular medium that elicited immune and protective responses [11]. In addition, interleukin-10 secreted by *L. lactis *was shown to have biological activity in mice [17]. Dieye et al. 2003 [18] also reported that the presentation of infectious bursal disease virus antigens (VP2) utilizing *Lactococcus *as a delivery vehicle showed a partial protection of the cell wall bound Nuc-VP2 against proteolysis as opposed to secreted Nuc-VP2. Recently, Ramasamy et al. [19] reported their work on the immunogenicity of a malaria parasite antigen displayed by *Lactococcus lactis *in oral immunizations. However, lactococcal system for vaccine delivery is hindered due to low levels of expression recombinant protein in *Lactococcus* and the use of antibiotic markers in recombinant *Lactococcus* often makes the bacteria resistance to antibiotics. In addition, we cannot control the expression of antigens when we directly make recombinant* Lactococcus* for vaccine delivery. We need alternative strategy to overcome some of these problems associated with* Lactococcus*. We selected *E. coli *as expression host to produce the fusion proteins (antigen/anchor) to overcome low expression associated with *Lactococcus. E. coli* have a number of commercially established high protein expression vectors and *E. coli* can easily be grown in a bioreactor and the recombinant proteins can be purified using simple purification systems such as fast protein liquid chromatography (FPLC). In addition, specific concentration of proteins (antigen) can be calculated, mixed with the appropriate number of *L. lactis*, where we can control dose vaccine by controlling a number of antigens and *Lactococcus* molecules. Since recombinant plasmids are not introduced into *Lactococcus* which eliminates antibiotic marker as selective pressure, this, therefore eliminates the worry of antibiotic resistant genes contaminating the environment when using recombinant vaccines. A number of advantages with *E. coli* make them an attractive host for the expression of fusion proteins (EV71 epitopes fused with cell wall binding domain of AcmA). AcmA is an autolysin which plays a key role in* Lactococcus* growth and propagation. AcmA naturally expressed in *Lactococcus* and expressed AcmA travels to cell wall and binds to the cell wall. Once it binds to cell, it starts the lysis of cell wall to release intracellular proteases into the media to digest the proteins into micronutrients which requires their cell survival. We utilized this natural phenomenon of the AcmA protein for the cell wall binding of EV71 epitopes. Cell wall binding domain of AcmA has three repeated regions of lysin motif (LysM) domains. The LysM domain is about 40 amino acids long and present in a number of surface associated proteins in a wide range of bacteria. The LysM domain has a **β** *αα* **β**structure and conserved asparate or glutamate in this shallow groove assumed to be involved in the binding with peptidoglycan and the mechanism of AcmA binding to cell wall was unknown. 

In this study, the N-terminal fragments of VP1 of EV71 were subcloned into pSVac to allow for the expression of C-terminal fusion proteins. The sequences of VP1_1-67aa_ and VP1_35-100aa_ at the N-terminal region of the VP1 protein of EV71 were chosen as antigens to be displayed on *Lactococcus*. VP1 protein of EV71 has high immunogenicity and antigenicity [20–22], and it has been a major candidate for the development of vaccines [20]. The studies by Hovi and Roivainen [23] showed that a highly conserved region of 42–52 amino acids close to the N-terminus of VP1 was involved in immunogenicity and that antibodies against this region can be used as a group reagent recognizing Enteroviruses. Peptide antibodies against 42–52 amino acid motif were shown to be capable of precipitating purified poliovirus particles, indicating that this region is exposed and involved in immunogenicity [24]. To create an N-terminal epitope for surface display, VP1 gene was truncated into VP1_1-201nt_ and VP1_103-300nt_ regions. The VP1_1-201nt_ region represented amino acids 1 to 67, and VP1_103-300nt_ region represented amino acid sequences 35 to 100, both from the N-terminal. The truncation of VP1 protein was done to increase the solubility of fusion protein and keep the structure small to avoid the possibility of masking the cell wall binding domains of AcmA. In this vector construct, the foreign genes were cloned upstream of the *acmA *gene fragment, thus allowing for a free C-terminal fusion for binding to the cell wall surface of Lactococcal cells. The AcmA/VP1_1-67aa_ and AcmA/VP1_35-100aa_ fusion proteins were then purified and targeted to the cell surface of *L. lactis*, and the recombinant *Lactococci *was used to immunize BALB/c mice by oral administration. Both the VP1_1-67aa_ and VP1_35-100aa_ could be docked onto the surface of *L. lactis*.

The AcmA repeat cell wall anchor has been previously used for the surface expression of the *Bacillus licheniformis *alpha-amylase and *E. coli *beta-lactamase [25], and the mechanism by which the acmA encoded attachment domains interact with the cell wall components has been suggested to be covalent in nature [26, 27]. Our main concern was the folding and stability of the fusion proteins after they were expressed in *E. coli *and purified. The expression of foreign genes in *E. coli *has been well documented [28, 29]. Observations from immunofluorescence studies showed that the purified AcmA proteins from *E. coli* cells had maintained their capability to anchor onto the surface of *Lactococcus *cells and are stably docked for at least 5 days [13]. Free proteins that may have been detached from the Lactococcal cell carrier presumably will not be able to survive the gastrointestinal tract to render any immunological reaction [30, 31]. Immunogenicity results indicated an immunogenic reaction in the test mice where the production of specific antibodies against VP1_1-67aa_ and VP1_35-100aa_ was observed by ELISA and Western blot analyses. The VP1_1-67aa_ and VP1_35-100aa_ antigens carrying *Lactococcus *represents the first step towards the development of a new strategy for vaccination against EV71 and perhaps other viral infections. Such a delivery system, utilizing lactic acid bacteria for oral administration of vaccine through food and water, would be very attractive because of its safety, low cost, and nonimmunosuppressing properties. In conclusion, a cell surface display system in which the AcmA cell wall binding protein of *L. lactis *was used as an anchoring motif was studied. Fusion proteins of up to 79 amino acids long were successfully displayed on the *L. lactis *outer membrane. Furthermore, the strains developed in this study were shown to be capable of inducing immunogenicity in orally fed mice. We believe that the method of protein docking utilized in this study will allow for more flexible presentations of short peptides and polypeptides on the surface of *L. lactis *to be useful as a delivery vehicle.
